# Determination of the residue levels of nicarbazin and combination nicarbazin-narasin in broiler chickens after oral administration

**DOI:** 10.1371/journal.pone.0181755

**Published:** 2017-07-27

**Authors:** Arina Lopes de Lima, Fabiano Barreto, Renata Batista Rau, Guilherme Resende da Silva, Leonardo José Camargos Lara, Tadeu Chaves de Figueiredo, Débora Cristina Sampaio de Assis, Silvana de Vasconcelos Cançado

**Affiliations:** 1 Departamento de Tecnologia e Inspeção de Produtos de Origem Animal, Escola de Veterinária, Universidade Federal de Minas Gerais (UFMG), Belo Horizonte, Minas Gerais, Brazil; 2 Laboratório Nacional Agropecuário (LANAGRO-RS), Ministério da Agricultura, Pecuária e Abastecimento, Porto Alegre, Rio Grande do Sul, Brazil; National Veterinary Research Institute, POLAND

## Abstract

The depletion times of the anticoccidial nicarbazin administered individually and of nicarbazin and narasin administered in combination were evaluated by determining the presence and levels of 4,4'-dinitrocarbanilide (DNC), the marker residue for nicarbazin, and narasin residues in the muscle tissues of broiler chickens subjected to a pharmacological treatment. A high-performance liquid chromatography-tandem mass spectrometry (HPLC-MS/MS) method was used. The results showed the presence of all anticoccidial residues; however, the DNC levels were higher when the nicarbazin was administered individually than when it was used in association with narasin throughout the experimental period. After six days of withdrawal, the DNC level following nicarbazin administration alone was lower than the maximum residue level (MRL) of 200 μg kg^-1^. However, when the nicarbazin was co-administered with narasin, the concentrations of DNC were lower than the MRL after four days of withdrawal. These results may be justified because the dosage of nicarbazin, when administrated individually, is greater than when it is used in combination with narasin. The levels of narasin were lower than the MRL of 15 μg kg^-1^ throughout the evaluation period. It was concluded that nicarbazin is rapidly metabolized from the broiler muscles up to six days of withdrawal since the DNC levels were lower than the maximum residue level (MRL) and the concentrations of narasin were lower than the MRL throughout the evaluation period.

## Introduction

Nicarbazin is a non-ionophoric synthetic complex composed of an equimolar amount of 4,4’-dinitrocarbanilide (DNC) and 2-hydroxy-4,6-dimethyl pyrimidine (HDP). It is authorized as a coccidiostat feed additive for individual use in chickens for fattening or in combination with the ionophore narasin, which has a monobasic carboxylic acid structure containing five cyclic ether rings [1]. Nicarbazin is well absorbed from the gastrointestinal tract and widely distributed in the body after oral administration. The components HDP and DNC have different pharmacokinetic characteristics; DNC is used as marker residue for evaluating food safety because it occurs at higher concentrations than HDP. DNC concentrates in the liver and kidneys, being excreted primarly in the faeces, while HDP is excreted in urine [2,3]. Narasin is poorly absorbed in the gastrointestinal tract and rapidly metabolized, being largely excreted in the bile, leading to the excretion of a high proportion of the administered dose via the feces [4].

Although nicarbazin is considered an effective and reliable anticoccidial product [5], it is known to produce heat-induced side effects in broilers [6]. Heat stress may increase mortality in nicarbazin-treated birds at an early age, from 18 to 29 days of age [7]. Thus, in warmer countries, which commonly use non-climatized rearing sheds, the use of nicarbazin alone is commonly recommended to only 21 days of age. However, the use of nicarbazin and narasin in combination reduces the required dosage of each one, thereby minimizing the side effects of nicarbazin. A nicarbazin-narasin combination may be used in broiler chickens reared in non-climatized sheds until 28 days of age.

The use of anticoccidial agents in poultry production may be justified because broilers are typically maintained in rearing sheds at high stocking densities that facilitate environmental re-infection of the birds and challenge the control of coccidiosis [8]. However, the use of such medications may lead to residues in food products from animal origin. These residues have significant human food safety implications, especially when there is non-compliance with the withdrawal period determined for each medication. Therefore, maximum residue limits (MRLs) for veterinary drug residues in foods of animal origin have been established. The Codex Alimentarius Commission established the classification of pharmacologically active substances and their respective MRLs in foodstuffs of animal origin; MRLs for nicarbazin and narasin in chicken muscle are 200 μg kg^-1^ and 15 μg kg^-1^, respectively [9]. To ensure compliance with this regulation, sensitive and specific analytical methods, such as liquid chromatography-tandem mass spectrometry (LC-MS/MS), should be used [10].

Several reports have been published regarding the development and validation of analytical methods by LC-MS/MS for the study of anticoccidial residues in avian muscle tissue [11–13]. However, studies on the depletion time of nicarbazin and narasin in broiler chickens using quantitative and confirmatory methodologies, such as LC-MS/MS [14], are lacking. The determination of appropriate withdrawal periods for drugs or feed additives used in poultry production based on the scientific study of depletion times has economic impacts and influences breeding management, especially in the final stages of broiler production. The effects on management promote proper animal treatment and the slaughter of animals in compliance with the required period for the elimination of each substance and its residues in the animal's body.

Thus, the purpose of the present work was evaluate the depletion time of the anticoccidial nicarbazin used individually and in a nicarbazin-narasin combination in broiler chickens. Using a high-performance liquid chromatography-tandem mass spectrometry (HPLC-MS/MS) method, we investigated the presence and levels of nicarbazin and narasin residues in the muscle tissues of broiler chickens subjected to a pharmacological treatment.

## Materials and methods

### Experimental animals

Three hundred sixty 1-day-old Cobb chicks were housed in pens containing 60 birds each (10 birds/m^2^) with *ad libitum* access to water and feed, in order to simulate the commercial raising conditions. The chickens were randomly allocated into three experimental groups, labeled A through C, containing 120 birds each. Group A was the untreated control group and received non-medicated feed. Chickens from group B received medicated feed containing nicarbazin from 1 to 21 days of age, and the birds from group C were fed medicated feed containing a nicarbazin-narasin combination from 1 to 28 days of age. After these periods, non-medicated feed was offered to all of the birds until the end of the experimental period. The dosage added to feed was established according to manufacturer’s instructions, i.e., 125 mg of nicarbazin per kg of feed (group B) and 50 mg of narasin + 50 mg of nicarbazin per kg of feed (group C). However, to ensure that the recommended dosage was provided to the broilers, the feed was analyzed by LC-MS/MS, according to the methodology described by Cronly et al. [15], to assess the actual amount of nicarbazin and narasin present. The level of nicarbazin found in the feed provided to group B was 125.65 mg kg^-1^, and 53.67 mg kg^-1^ of narasin and 48.83 mg kg^-1^ of nicarbazin were detected in the feed of group C. No anticoccidials were detected in the feed provided to group A.

Six birds of each group were slaughtered on different days to collect muscles of thigh and breast. Chickens from group A were slaughtered at 14, 21, 27, 28 and 34 days of age. Birds from group B were slaughtered at 14, 21, 22, 23, 25, and 27 days of age, and birds from group C were slaughtered at 14, 21, 28, 29, 30, 32 and 34 days of age. The samples were individually collected and stored at -20°C for HPLC-MS/MS analyses, according to the methodology previously validated by Barreto et al. [16].

This study was carried out in strict accordance with the recommendations of the National Council for the Control of Animal Experimentation (CONCEA) at the Brazilian Ministry of Science and Technology and Innovation (MCTI). The protocol was approved by the Ethics Committee in Animal Experimentation at the Universidade Federal de Minas Gerais (UFMG) (Permit Number: 300/2014). Broiler chickens were euthanized according to the principles of humane slaughter and properly stunned using the method of electrical stunning before bleeding to minimize suffering.

### Chemicals and reagents

The analytical standards dinitrocarbanilide (DNC) and narasin (NAR) were purchased from Sigma-Aldrich (St. Louis, MO, USA). Dinitrocarbanilide-d8 (DNC-d8) and decoquinate-d5 (Decq-d5) were used as internal standards for determination of DNC and NAR, respectively, and were purchased from Witega (Berlin, Germany).

Acetic acid, ammonium acetate, methanol and hexane from J.T. Baker (Phillisburg, USA); acetonitrile and dimethyl sulfoxide from Merck (Darmstadt, Germany); and chloroform from Sigma-Aldrich, were used. All of the solvents were of HPLC grade or superior, and the reagents were of analytical grade. Ultrapure water was obtained from a Milli-Q System (Millipore, Bedford, MA, USA).

### Standard solutions

Standard stock solutions of DNC, DNC-d8, NAR and Decq-d5 were prepared at a concentration of 1 mg mL^-1^ by dissolving DNC and DNC-d8 analytical standards in dimethyl sulfoxide, NAR standard in methanol and Decq-d5 standard in chloroform.

The standard stock solutions were diluted in acetonitrile, resulting in a working mixed standard stock solution with a final concentration of 5 μg mL^-1^ of DNC, DNC-d8 and Deqc-D5 and 1.5 μg mL^-1^ of NAR.

### Extraction procedure

Prior to sample analysis, it was made a pool of breast and thigh muscles. The analytes were extracted from 2 g samples of muscle that had been weighed in 50 mL polypropylene centrifuge tubes. The samples were added to 10 mL of acetonitrile and homogenized with Ultra-Turrax^®^ (IKA, Wilmington, NC, USA). Then, the tubes were agitated for 20 min and centrifuged (4000 x g) at 5°C for 10 min. The supernatant was transferred to 15 mL polypropylene centrifuge tubes, stored at -15°C for 60 min and then centrifuged at 4000 x g. The supernatant was transferred to 50 mL polypropylene tubes and evaporated at 45°C under nitrogen flow. After this step, 2 mL of hexane and 2 mL of water:acetonitrile (1:1 v/v) were added, and the tubes were agitated at 80 rpm for 10 min. Then, 1 mL of the obtained extract was reserved in a vial for injection.

### Instrumentation

The experiments were performed in an Agilent HPLC system coupled to an AB Sciex API 5500 QTRAP triple quadrupole mass spectrometer (AB Sciex, Foster City, CA, USA). A Poroshell 120 EC-C18 (3.0 x 50 mm, 2.7 μm) column (Agilent, Milford, MA, USA) with a C18 pre-column (Phenomenex, CA, USA) was used for the chromatographic separation. The column temperature was set at 40°C. Mobile phase A consisted of water with 0.5% acetic acid and 1 mM ammonium acetate, and mobile phase B consisted of acetonitrile with 0.5% acetic acid and 1 mM ammonium acetate. The flow rate was set at 0.5 mL min^-1^. Initial conditions were set at 98% A with a linear gradient from 98% A to 5% A for 1 min. Then, 5% A was held for 8 min with an immediate return to 98% A from 9 min to 10 min. The total run time for each injection was 12 min, and the injection volume was 4 μL.

The operational conditions of the mass spectrometer were established by direct infusion of the standards. Two MRM transitions were established and monitored for each analyte (Table 1). Nitrogen was used as collision gas at 55 psi. Ion source gas 1 (GS1) and ion source gas 2 (GS2) were used at 55 psi and 20 psi, respectively. The ion source was operated in the positive and negative mode. Capillary voltage was set at 5.5 kV in the positive mode and -4.5 kV in the negative mode. The temperature of the source was set at 500°C.

**Table 1:** Transitions, relative intensity of the ions and MS/MS parameters used for each analyte.

Analyte	Precursor ion	Product ion	EP^a^ (V)	CE^b^ (V)	DP^c^ (V)	CXP^d^ (V)	Dwell time (s)	RT^e^ (min)
DNC	301.0	137.0	-10	-18	-50	-13	0.050	3.37
	301.0	106.8	-10	-50	-55	-15	0.050	3.37
DNC-d8	308.9	141.2	-10	-22	-30	-9	0.050	3.37
	308.9	111.0	-10	-50	-75	-9	0.050	3.37
Decq-d5	423.4	377.2	10	33	236	24	0.050	4.10
	423.4	205.0	10	55	236	16	0.050	4.10
NAR	782.4	747.3	10	27	106	20	0.050	5.95
	782.4	729.3	10	31	106	14	0.050	5.95

^a^EP—entrance potential.

^b^CE—collision energy.

^c^DP—declustering potential.

^d^CXP—collision cell exit potential.

^e^RT—retention time.

### Statistical analysis

To assess the depletion times of the anticoccidials, DNC levels were subjected to ANOVA and compared using the Tukey test at a 5% significance level. The withdrawal period was established based on the MRL of each drug, taking into consideration the 99% tolerance limit with 95% confidence interval [17].

## Results and discussion

Residues of the anticoccidials nicarbazin and narasin were not detected in any of the samples from the control group (group A). Among these samples, only the 1^st^ and 2^nd^ transitions of the internal standards Decq-d5 and DNC-d8 were detected, indicating that there was no contamination of the feed during the treatment.

In the group of animals that received nicarbazin-medicated feed, the highest concentrations of DNC were found at 14, 21 and 22 days of age (P<0.05) (Table 2).

**Table 2:** Mean concentrations of DNC analyzed by HPLC-MS/MS method in the muscle matrix from broilers that received nicarbazin-medicated feed according to chicken day of age.

Day of age	Days of withdrawal	DNC concentration (μg kg^-1^)
14		5353.33 ± 428.42 a
21	0	5070.00 ± 1090.54 a
22	1	3431.67 ± 594.45 a
23	2	1220.17 ± 372.63 b
25	4	212.23 ± 135.57 c
27	6	34.80 ± 16.40 d

Means followed by different letters differed significantly according to the Tukey test (P<0.05).

**Fig 1:**
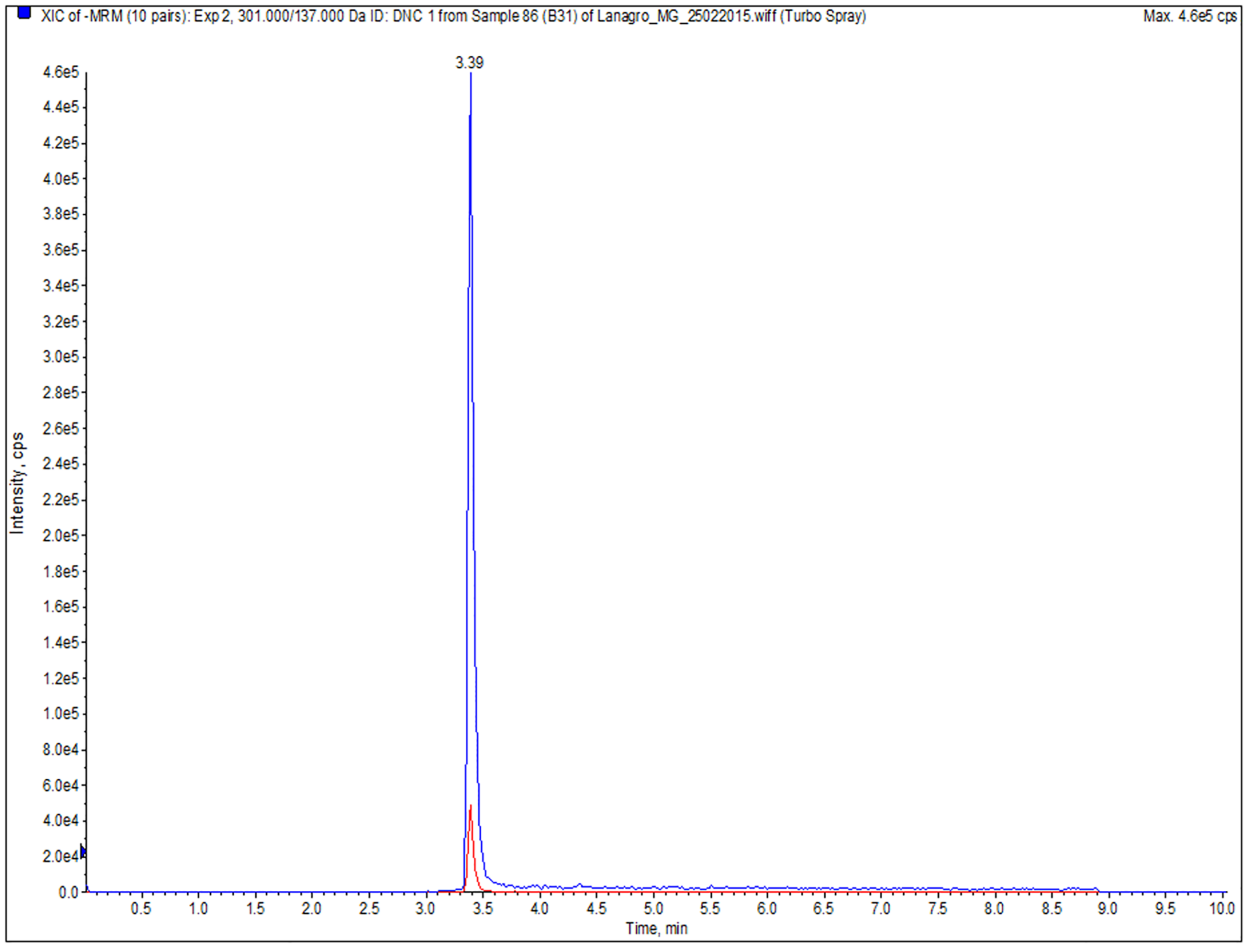
Chromatogram of a muscle sample from group B collected at 25 days of age demonstrating the presence of the 1^st^ and 2^nd^ transitions of DNC.

**Fig 2:**
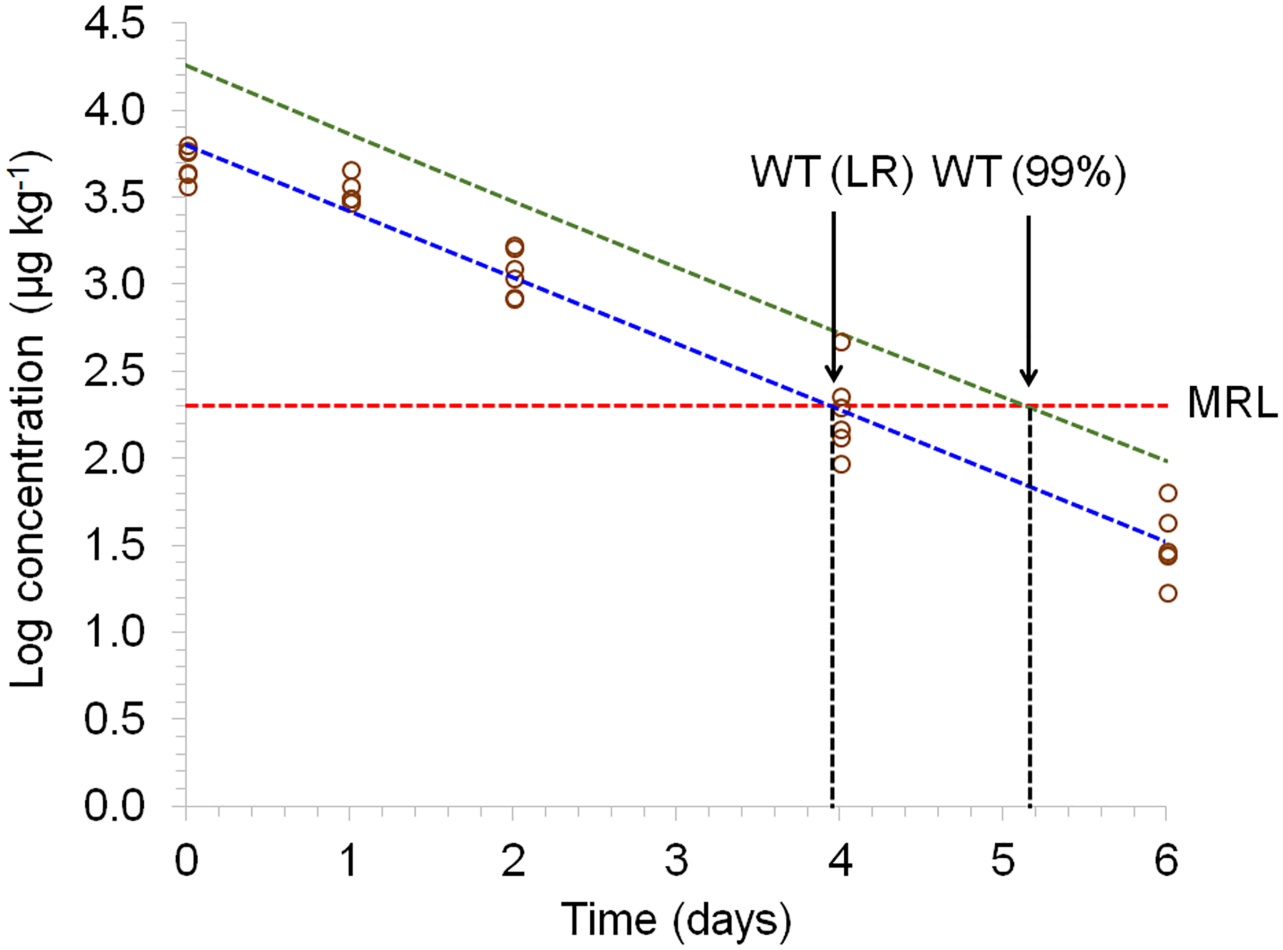
Representative semi-logarithmic plot of residue concentration in muscle from broiler chickens subjected to a pharmacological treatment with nicarbazin (group B) for DNC vs. time for 99% tolerance level. Circles represent the residue concentration of DNC for an individual animal. The straight line and the curve represent the regression line (LR) and its 99% tolerance limit with 95% confidence interval, respectively. The withdrawal period calculation based on the MRL (200 μg kg^-1^) is plotted with a vertical line.

The DNC levels found at 14 and 21 days of age were similar because during this period the chickens received medicated feed containing nicarbazin. After 21 days of age, non-medicated feed was provided, and a gradual decrease in DNC level was observed. Residue concentrations of DNC that were higher than the MRL (200 μg kg^-1^) were found in muscle samples until 25 days of age (Fig 1), i.e., four days after the end of treatment. However, considering the 99% tolerance limit with 95% confidence interval, the withdrawal time of DNC was six days after cessation of the medication, when the DNC levels found were lower than the MRL (Fig 2). In the UK, nicarbazin is licensed for use as feed additive in the production of broiler chickens with a withdrawal period of 9 days [18]. Nevertheless, according to the European Food Safety Agency (EFSA), the withdrawal period recommended for nicarbazin use in broiler chickens is one day [2]; however, this withdrawal period was not set considering the MRL established by the Codex Alimentarius [9] (200 μg kg^-1^), but a much higher MRL, i.e., 4 mg kg^-1^ for the muscle tissue. Therefore, based on the results found, if the MRL adopted by the Codex Alimentarius is considered, only one day of drug withdrawal is not sufficient for total elimination of DNC residues from muscle tissue, as DNC levels higher than 200 μg kg^-1^ were found up to six days after cessation of the medication.

The anticoccidial nicarbazin is well absorbed from the gastrointestinal tract and distributed broadly to tissues after oral administration. Nicarbazin is rapidly split into its components HDP and DNC, which have different pharmacokinetic characteristics. DNC occurs at higher concentrations than HDP and concentrates in the liver and kidneys, and is excreted primarly in the faeces, while HDP is excreted in urine; therefore, it is used as marker residue for evaluating food safety [2,3]. Depletion of DNC was studied in hen and broiler tissues and the concentrations in the liver were much higher than those observed in breast muscle samples [14,18]. Danaher et al. carried out a survey to evaluate the occurrence of DNC residues in poultry carcasses and found no muscle samples with concentrations higher than the DNC MRL, while 12.1% of liver samples contained residues at levels above 200 μg kg^-1^. Thus, the liver is the most appropriate matrix for monitoring DNC residues, because it is the matrix in which residues persist at highest levels [19]. However, despite the differences in pharmacokinetic characteristics and concentrations found in different tissues, the MRL established by Codex Alimentarius is the same for chicken muscle, liver, kidney and fat/skin, i.e. 200 μg kg^-1^ [9]. Although the liver is the targeted tissue for monitoring DNC residues, only muscle samples are tested in Brazilian National Plan for Control of Residues and Contaminants, due to the availability of a validated methodology for analysis of this matrix.

Due to the heat-induced side effects produced by nicarbazin in broiler chickens, its use is often recommended only until 21 days of raising. As a result, although high levels of DNC are detected during the treatment period of the birds, they are rarely found in the meat when the chickens reach the age of slaughter. However, in some cases, pharmacological treatment may involve inappropriate durations or dosages or lead to the contamination of non-medicated feed, leading to the presence of residues in chicken meat at concentrations above the MRL. A number of studies were developed to identify the possible causes of nicarbazin residues in broiler chicken tissues and have been demonstrated that DNC residues may occur in chickens fed withdrawal ration contaminated with low concentrations of nicarbazin [17,20–21]. The housing method may also influences DNC level in broiler tissues, because residual tissue concentrations in birds housed on deep litter was higher than those of broilers housed on wire flooring, due to faecal recycling [18]. The recycling of DNC from litter was also suggested by Olejnik et al. because the litter consumption by the birds and the concentrations of DNC in gastric content [14].

**Fig 3:**
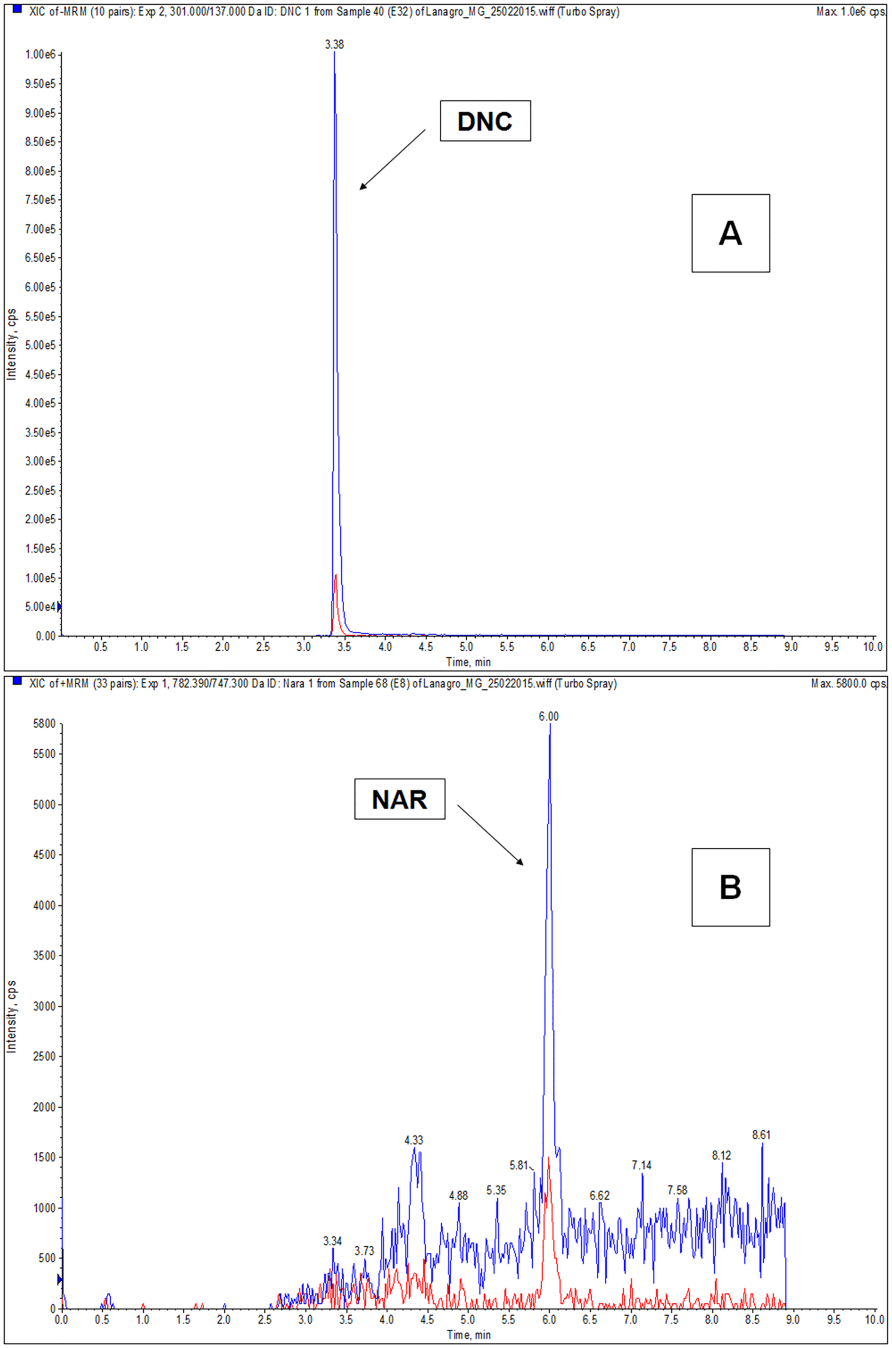
Chromatogram of a muscle sample from group C collected at the last day of administration (28 days of age) demonstrating the presence of the 1^st^ and 2^nd^ transitions of DNC (A) and NAR (B).

In the group of chickens that received medicated feed with a nicarbazin-narasin combination (group C), residues of NAR were detected in 11 of 42 samples analyzed. However, only 5 samples showed concentrations higher than LOQ of the method (3.75 μg kg^-1^), and no positive sample showed a concentration higher than the MRL of 15 μg kg^-1^ established by Codex Alimentarius [9] (Table 3). A chromatogram of a positive muscle sample, collected at the last day of administration, is shown in Fig 3. These results suggest that narasin is poorly absorbed in the gastrointestinal tract and rapidly metabolized, as the concentrations found in the muscle tissue, even during the treatment period were low. Moreover, narasin is largely excreted in the bile, leading to the excretion of a high proportion of the administered dose via the feces [22].

**Table 3:** Mean concentrations of NAR residues analyzed by HPLC-MS/MS in the broiler chicken muscle matrix according to chicken day of age.

Day of age	Days of withdrawal	Narasin concentration (μg kg^-1^)		
		Min	Max	Median
14		ND	<LOQ	ND
21		ND	4.32	<LOQ
28	0	ND	5.81	<LOQ
29	1	ND	3.78	ND
30	2	ND	<LOQ	ND
32	4	ND	ND	ND
34	6	ND	<LOQ	ND

Min, minimum values; Max, maximum values; ND, not detected: lower than the limit of detection of the method (1.87 μg kg^-1^); LOQ, limit of quantification (3.75 μg kg^-1^).

In contrast to narasin residues, residues of DNC were found at high concentrations in the group of chickens treated with a nicarbazin-narasin combination. The highest values (P<0.05) were found at 14, 21 and 28 days of age, i.e., only during the treatment period (Table 4). At 32 days of age, the DNC concentrations were lower than the MRL of this feed additive and at 34 days of age, residues of narasin were not detected, as all samples showed concentrations lower than LOD of the method (6.25 μg kg^-1^).

**Table 4:** Mean concentrations of DNC analyzed by HPLC-MS/MS in the muscle matrix from broilers that received medicated feed with a nicarbazin-narasin combination according to chicken day of age.

Days of age	Days of withdrawal	DNC concentration (μg kg^-1^)
14		2863.33 ± 313.86 a
21		2748.33 ± 408.28 a
28	0	2288.33 ± 375.79 a
29	1	1103.83 ± 433.77 b
30	2	418.66 ± 154.69 c
32	4	35.63 ± 26.50 d

Means followed by different letters differed significantly according to the Tukey test (P<0.05). ND, not detected: lower than the limit of detection of the method (6.25 μg kg^-1^).

**Fig 4:**
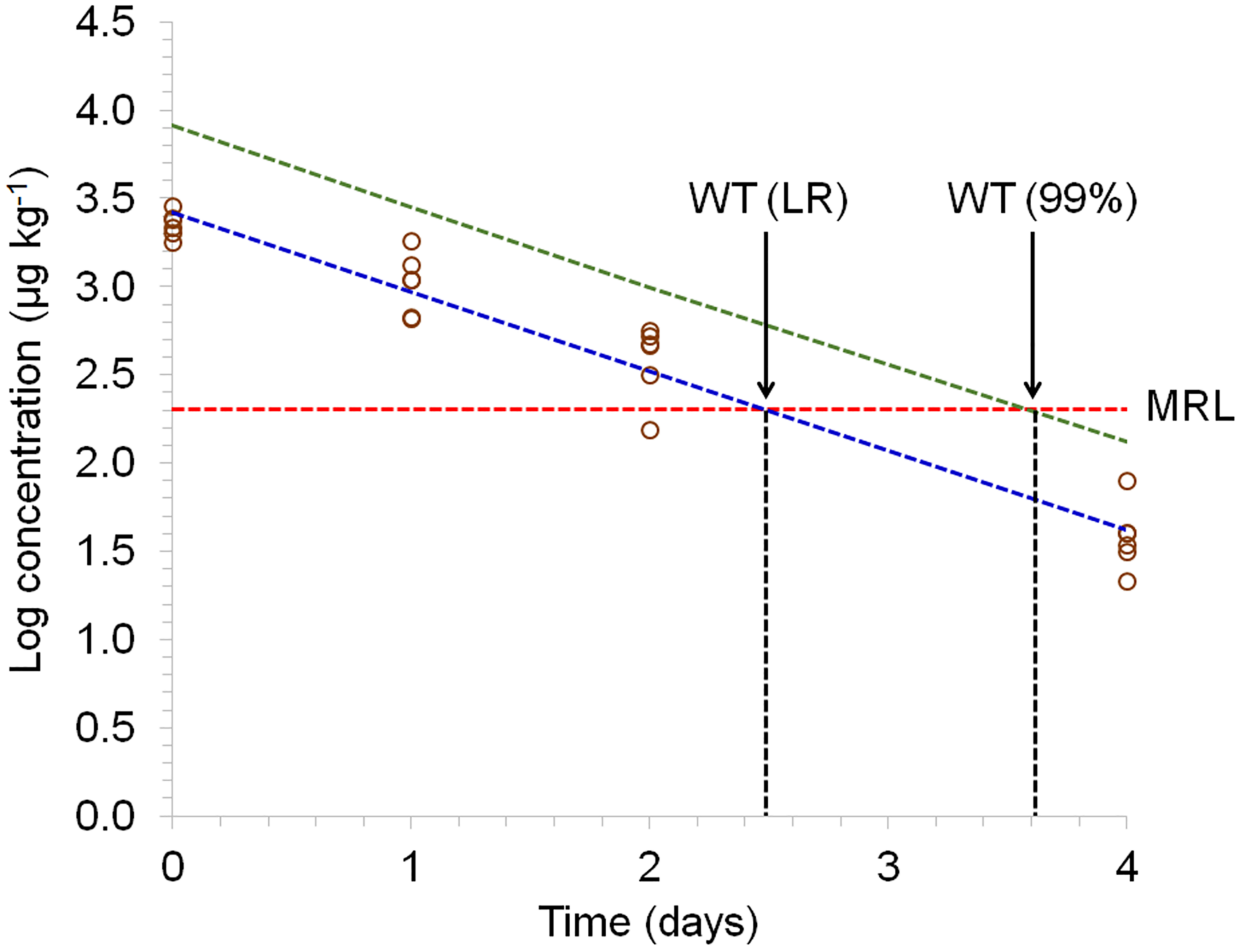
Representative semi-logarithmic plot of residue concentration in muscle from broiler chickens subjected to a pharmacological treatment with nicarbazin + narasin (group C) for DNC vs. time for 99% tolerance level. Circles represent the residue concentration of DNC for an individual animal. The straight line and the curve represent the regression line (LR) and its 99% tolerance limit with 95% confidence interval, respectively. The withdrawal period calculation based on the MRL (200 μg kg^-1^) is plotted with a vertical line.

The characteristics of DNC in this group were similar to those observed in group B. The mean concentrations at 14, 21 and 28 days of age (during the pharmacological treatment period) were similar (2.863 μg kg^-1^, 2.748 μg kg^-1^, 2.288 μg kg^-1^), and a gradual reduction in DNC level was observed after this period, when non-medicated feed was provided for the chickens (Fig 4). However, the DNC levels of group C were lower than those of group B because the dosage of nicarbazin required to achieve therapeutic effects is lower when this drug is used in combination with narasin.

Narasin is an ionophore coccidiostat that is rapidly metabolized and poorly absorbed in the gastrointestinal tract, being excreted via faeces. These pharmacokinetic characteristics may be related to the lower MRL values, established by Codex Alimentarius, for narasin than nicarbazin, whereas the residue tolerance limits for narasin are 15 μg kg^-1^ for muscle and kidney and 50 μg kg^-1^ for liver and fat, the MRL for nicarbazin was set at 200 μg kg^-1^ for all matrices evaluated in chicken tissues [9]. The low possibility of occurrence of its residues is supported by the results of depletion studies of narasin [14] or other ionophore antibiotics in chickens, such as salinomycin and monensin [22,23]. Salinomycin, the substance with a chemichal structure and properties most similar to narasin, was determined by ELISA and the results demonstrated its presence in the liver at low concentrations [22]. Residues of monensin were also found at low concentrations and depleted rapidly [23].

Although concentrations of narasin were found below the MRL throughout the experimental period and a zero-day withdrawal period was considered appropriate to this substance, as it was used in combination with nicarbazin, the feed additive with the longer depletion time should be considered for the establishment of the withdrawal period. Thus considering that DNC residues were found at concentrations lower than MRL only at 32 days of age (four days after cessation of medication), this minimum period should be considered to occur the elimination of its residues from the muscle tissues. In addition, it should be considered that for other matrices, such as the liver, kidney or skin/fat, the depletion time may be longer.

## Conclusion

This depletion study demonstrated that when the nicarbazin is administrated alone, the withdrawal time is six days. However, when this drug is co-administered with narasin the withdrawal time for nicarbazin is four days and for the anticoccidial narasin, a zero-day withdrawal period is considered appropriate, since the levels found were lower than the maximum residue level (MRL) at all time points of evaluation.
